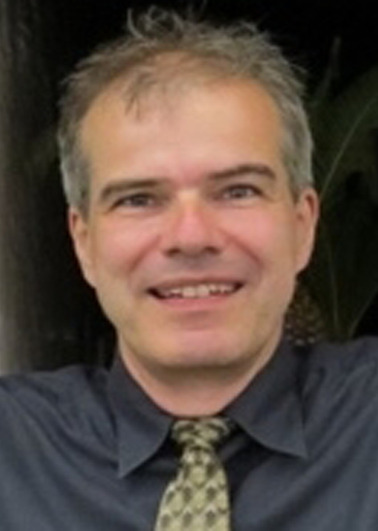# Visible‐Light‐Induced Homolysis of Earth‐Abundant Metal‐Substrate Complexes: A Complementary Activation Strategy in Photoredox Catalysis

**DOI:** 10.1002/anie.202100270

**Published:** 2021-06-18

**Authors:** Youssef Abderrazak, Aditya Bhattacharyya, Oliver Reiser

**Affiliations:** ^1^ Institut für Organische Chemie Universität Regensburg Universitätsstrasse 31 93053 Regensburg Germany

**Keywords:** 3d transition metals, dissociative ligand-to-metal charge transfer, inner-sphere electron transfer, photoredox catalysis, visible-light-induced homolysis

## Abstract

The mainstream applications of visible‐light photoredox catalysis predominately involve outer‐sphere single‐electron transfer (SET) or energy transfer (EnT) processes of precious metal Ru^II^ or Ir^III^ complexes or of organic dyes with low photostability. Earth‐abundant metal‐based M^n^L_n_‐type (M=metal, L_n_=polydentate ligands) complexes are rapidly evolving as alternative photocatalysts as they offer not only economic and ecological advantages but also access to the complementary inner‐sphere mechanistic modes, thereby transcending their inherent limitations of ultrashort excited‐state lifetimes for use as effective photocatalysts. The generic process, termed visible‐light‐induced homolysis (VLIH), entails the formation of suitable light‐absorbing ligated metal–substrate complexes (M^n^L_n_‐Z; Z=substrate) that can undergo homolytic cleavage to generate M^n−1^L_n_ and Z^.^ for further transformations.

## Graphical abstract

The use of earth‐abundant metal complexes in photoredox catalysis offers a complementary mode of activation for organic substrates through visible‐light‐induced homolysis (VLIH) of metal–substrate bonds. This Minireview describes different electronic transitions that occur in photoexcited metal–substrate complexes as well as recent advancements and future prospects in this area.

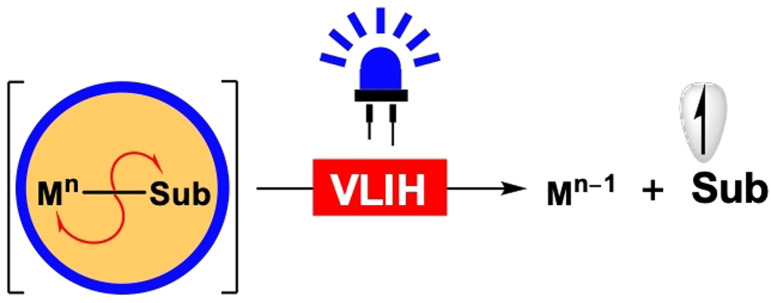

## 1 Introduction

The emergence and upsurge of visible‐light photoredox catalysis have made an ineradicable impact on contemporary organic synthesis in the last decade, providing access to unconventional reactivity profiles of small molecules by the efficient conversion of photonic into chemical energy.^[1]^ To date, the prevailing external chromophores used in such transformations are heavy transition‐metal catalysts with appropriate ligands such as Ru^II^‐ or Ir^III^‐polypyridyl complexes or metal‐free organic dye sensitizers as they possess long excited‐state lifetimes, strong absorption in the visible region of the electromagnetic spectrum, and the corresponding photoexcited states have high reduction or oxidation potentials.^[1c]^ The modes of action of the excited states of these photocatalysts are either single‐electron transfer (SET) or energy transfer (EnT) processes to generate various radical species complementary to common thermal two‐electron processes.^[2]^ However, organic dyes and heavy transition metal‐based complexes have downsides in terms of their low photostability^[2b]^ and adverse economic, biological, and environmental impacts,^[3]^ respectively. In this context, the exploration and exploitation of earth‐abundant and inexpensive 3d transition‐metal complexes as the next generation photocatalysts is rewarding from the perspectives of sustainability and large‐scale synthetic applicability.^[4]^ However, the wide application of such earth‐abundant metal complexes is greatly limited by their ultrashort excited‐state lifetimes (pico‐ to nanosecond range) compared to iridium‐ and ruthenium‐based photocatalysts (microsecond range), thus making the prospect of initiating bimolecular SET and EnT processes bleak.^[5]^

A few well‐designed copper complexes have been used as alternative photocatalysts that demonstrate distinct mechanisms involving electron transfer within the inner coordination sphere, thereby controlling reactions through their ligand environment.^[6]^ Other first‐row transition‐metal salts have been used as successful photocatalysts in isolated cases,^[5b]^ but more notably used as co‐catalysts in various photochemical coupling reactions.^[7]^

Complexes based on 3d transition metals generally possess a high degree of ligand‐substitution lability—a feature that impedes the attainment of favorable photoexcited‐state properties such as long lifetimes or photoluminescence.[^1c^, ^2a^] Nonetheless, this property can be creatively utilized for developing mechanistically distinct new photocatalytic processes, termed visible‐light‐induced homolysis (VLIH), complementary to the conventional/cooperative processes with coordinatively saturated and substitution‐inert heavy‐metal‐based photocatalysts.

**Figure 1:**
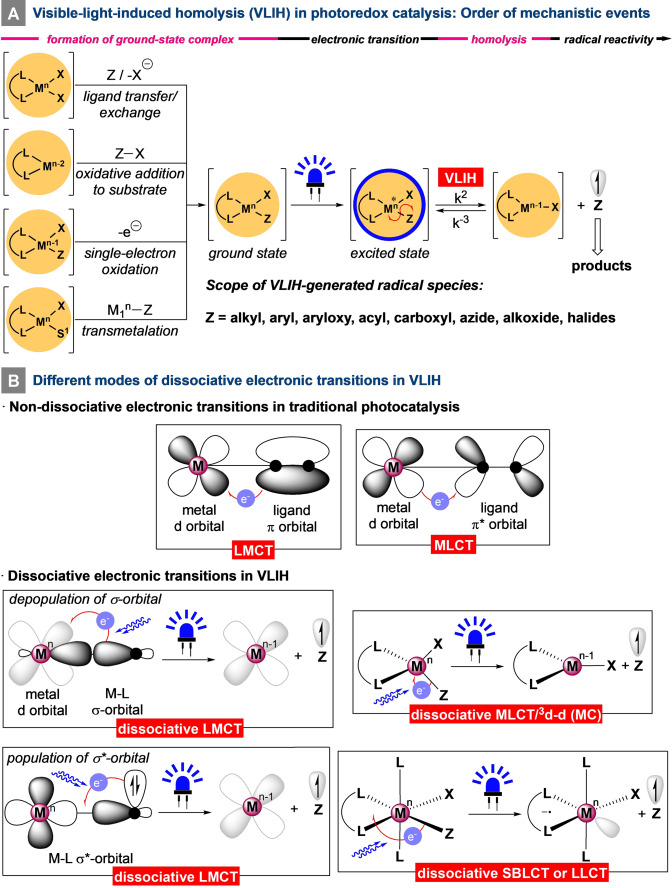
Mechanistic features of visible‐light‐induced homolysis (VLIH): Order of the mechanistic events and the modes of photoinduced electronic transitions.

The mechanism of VLIH proceeds through 1) the initial formation of the metal–substrate complex [L_*n*_M^*n*^(X)‐Z] from the electronic ground state of the metal complex and the substrate through ligand transfer/exchange, oxidative addition, single‐electron oxidation, or transmetalation; 2) photoexcitation of the metal–substrate complex to form [L_*n*_M^*n*^(X)‐Z]*; and 3) inner‐sphere redox processes through various metal‐complex‐specific electronic transitions that ultimately result in homolysis of the metal–substrate (M^*n*^‐Z) bond to generate the reduced metal species [L_*n*_M^*n*−1^(X)] and a radical species (Z^.^) from the substrate that is set for further transformations (Figure 1 A). The advantages of this strategy are the high chemoselectivity and site selectivity of the photochemical processes, as the targeted oxidation takes place solely at the transiently ligating atom or functional group, with other oxidation‐prone functionalities being left intact.

The key mechanistic event in the VLIH process is the homolytic cleavage of the metal–substrate (M−Z) bond, for which various inner‐sphere electronic charge‐transfer modes can be responsible. The traditional mononuclear heavy‐metal‐based photoactive complexes display metal‐to‐ligand charge transfer (MLCT) involving d→π*, ligand‐to‐metal charge‐transfer (LMCT) involving π→d, and intraligand (IL) transitions. In general, these charge‐separating transitions are nondissociative and do not result in the cleavage of the corresponding metal–ligand bonds. Therefore, the complexes can participate in various reversible outer‐sphere electron‐transfer processes without losing the integrity of their molecular structure.

In the majority of modern synthetic applications of VLIH, the LMCT electronic transition induces the desired homolysis of the M−Z bonds as a result of the ability of the M−Z complexes to absorb in the visible‐light region of the electromagnetic spectrum. However, these dissociative LMCT processes are inherently different from the nondissociative ones, as the dissociative processes tend to alter the electronic population of the σ/σ^*^ orbitals of covalent M−Z bonds either by depopulating the M−Z σ‐molecular orbital or by populating the M−Z σ*‐molecular orbital and usually engage metals to participate in the process from their high oxidation states^[8]^ (Cu^II^, Ni^III^, Fe^III^, Ce^IV^, Co^III^, etc; Figure 1 B).^[9]^

Nevertheless, VLIH does not always exclusively involve LMCT modes. Excitation of the metal–substrate complexes with visible light can result in other modes of electronic transitions that also induce homolysis of the M−Z bonds (Figure 1 B). In square‐planar [Ni^II^(^*t*‐Bu^bpy)(*o*‐Tol)Cl]‐type complexes, the VLIH events involve MLCT/^3^d‐d electronic transitions that result in cleavage of the Ni−aryl bond to generate aryl radicals and the corresponding Ni^I^ species.^[10]^ However, M(Sub)(CO)_3_(diimine)‐type complexes can demonstrate an alternate charge‐transfer mode from a σ‐bond to the ligand (SBLCT, σ→π*), thereby resulting in ligand reduction along with the generation of the radical (Z^.^) from the substrate (Figure 1 B).^[11]^ Although direct access to σπ* states is forbidden by spectroscopic transition rules, it can be generated by relaxation from the ^1^MLCT states. However, in some complexes with non‐oxidizable metal centers, access to ^1^MLCT states is also prohibited, rendering electrons prone to be directly transferred from the σ‐orbital of the M−Z bond to the antibonding orbitals of other ligands, which is regarded as a ligand‐to‐ligand charge‐transfer mode (L_σ_L_π*_CT).^[12]^

As mentioned earlier, most of the developments in newer synthetic methods reliant on the VLIH concept have been limited to LMCT transitions. However, the ever‐expanding development of spectroscopic and analytical techniques has led to the other types of electronic transitions being recognized as the effective cause of VLIH, which might open up opportunities in future developments for organic synthesis. In this Minireview, we discuss the advancements in the field of VLIH, their different mechanistic aspects based on charge‐transfer modes, and the prospects for its application in synthetic organic chemistry.

## 2 Copper

Copper(I)‐based complexes are rapidly emerging as capable visible‐light‐mediated photoredox catalysts that offer not only economic and ecological advantages but also otherwise inaccessible inner‐sphere mechanisms to enable challenging transformations.[^6^, ^9a^, ^9b^, ^13^] In contrast, there are only a handful of reports available for photocatalytic processes using Cu^II^ compounds. In the area of radical‐mediated organic reactions, chlorine radicals are attractive reactive species, partly because of their varied reactivity with different organic compounds and partly because of the easy availability of a wide array of earth‐abundant transition‐metal chloride salts as potential precursors. However, generating chlorine radicals from these salts by photochemical means is challenging, as the oxidation potential of the chloride anion is much higher (*E* ^o^(Cl^.^/Cl^−^)=+2.03 V vs. SCE in MeCN at 298 K)^[14]^ than the excited‐state oxidation potentials of commonly used photocatalysts.^[9c]^

**Scheme 1:**
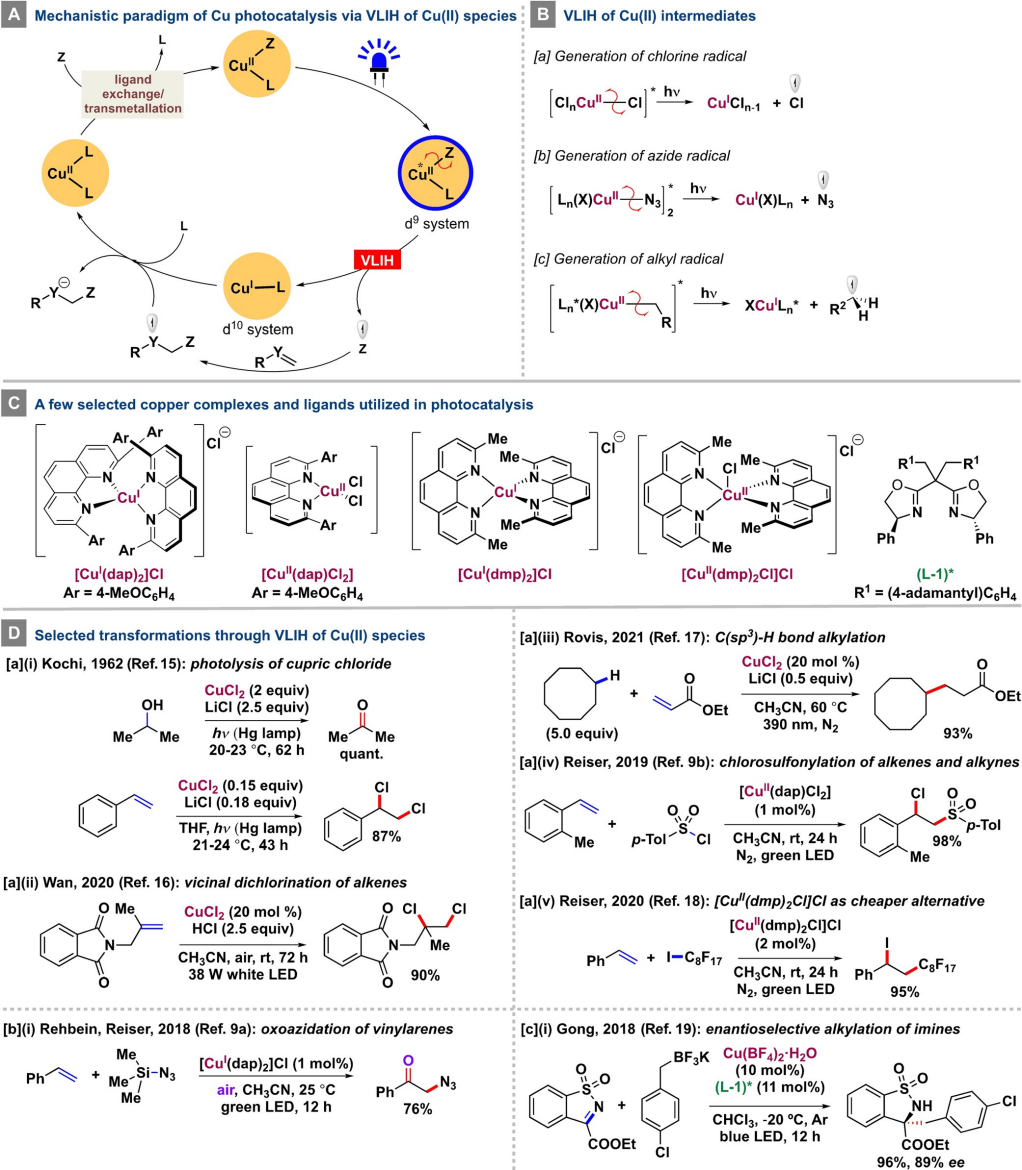
Mechanistic features of the VLIH of Cu^II^ species and selected transformations.

In 1962, Kochi observed the photolysis of cupric chloride (CuCl_2_; exists as a chlorocupric complex in organic media) to cuprous chloride (CuCl) and the chlorine radical under unfiltered radiation from a medium‐pressure mercury lamp at ambient temperatures.^[15]^ The observation could only be explained by a sequential process of ligand‐to‐metal charge transfer (LMCT) followed by homolysis of the Cu−Cl bond, thereby establishing one of the earliest examples of the VLIH principle with a copper salt. Subsequently, the photogenerated chlorine radical could be successfully exploited to perform different organic transformations, such as the quantitative oxidation of 2‐propanol to acetone or the formation of styrene dichloride from styrene in 87 % yield [Scheme 1 D, Eq. a‐i].

Capitalizing on Kochi's discovery, Wan and co‐workers developed a visible‐light‐induced vicinal dichlorination of olefins by directly using CuCl_2_ as a photoactive species without any exogenous ligand [Scheme 1 D, Eq. a‐ii].^[16]^ Although a combination of 20 mol % CuCl_2_ and 2.5 equiv hydrochloric acid as the chlorine source was required for effective dichlorination of unactivated olefins, 4.0 equiv CuCl_2_ alone were adequate to induce the same transformations for activated olefins upon irradiation with a 38W white LED (*λ*=390–760 nm). Very recently, the Rovis group achieved the selective C(sp^3^)−H alkylation and amination of feedstock alkanes with electron‐deficient olefins, such as acrylates and vinyl sulfones, in the presence of a catalytic amount of CuCl_2_ under irradiation with long‐wavelength UV light.^[17]^ The transformation proceeds by VLIH of an intermediate Cu^II^ species by LMCT to generate a chlorine radical which acts as a powerful hydrogen atom transfer reagent capable of abstracting strong electron‐rich C(sp^3^)−H bonds [Scheme 1 D, Eq. a‐iii].

In 2019, [Cu^II^(dap)Cl_2_] (dap=2,9‐bis(4‐methoxyphenyl)‐1,10‐phenanthroline) was used in a photochemical atom‐transfer radical addition (ATRA) reaction between sulfonyl chloride and olefins [Scheme 1 D, Eq. a‐iv].^[9b]^ In line with Kochi's proposal, VLIH of the LCu(II)−Cl bond generates the catalytically active LCu(I) species that initiates the reduction of sulfonyl chlorides. Improving on this concept, [Cu^II^(dmp)_2_Cl]Cl (dmp=2,9‐dimethyl‐1,10‐phenanthroline, Scheme 1 C) can be utilized as a more robust and economic photocatalyst compared with its dap variant, as the dmp ligand is inexpensive and commercially available [Scheme 1 D, Eq. a‐v].^[18]^ Direct spectroscopic evidence obtained from a follow‐up study in collaboration with the Castellano group^[8]^ has proved that cleavage of the L_*n*_Cu−Cl bond occurs in <100 fs and requires blue excitation into the Cl→Cu LMCT transition for the photochemical transformation of Cu^II^ to Cu^I^ and the generation of a reactive chlorine atom radical.

Activation of the LMCT state of Cu^II^X_2_‐type complexes endowed with suitable ligands other than halides by irradiation with visible light could also be expected to produce radicals (X^.^) by homolysis and these could initiate productive organic transformations (Scheme 1 A). In 2018, Reiser and co‐workers developed a photocatalyzed method based on Cu(dap)Cl_2_ (Scheme 1 C) for the synthesis of azido ketones from vinyl arenes and trimethylsilyl azide in air [Scheme 1 D, Eq. b‐i].^[9a]^ Mechanistically, the Cu^II^ complex undergoes ligand exchange with azide to generate a new LCu^II^N_3_‐bridged dimer, which upon VLIH forms an LCu^I^ species and an azido radical. The incipient azido radical can be intercepted by an alkene, followed by trapping of molecular oxygen. The rebinding of the O‐centered radical with LCu^I^ regenerates the LCu^II^ species, which releases the product and closes the catalytic cycle.

Shortly after this report, Gong and co‐workers developed the visible‐light‐induced copper(II)‐catalyzed enantioselective alkylation of imines [Scheme 1 D, Eq. c‐i],^[19]^ wherein a chiral Cu^II^‐bisoxazoline complex is alkylated through transmetalation from the corresponding alkyl trifluoroborate salt and, subsequently, VLIH generates an alkyl radical and a Cu^I^ intermediate. In a second catalytic cycle, this alkyl radical adds to a protected imine, which is activated by the same chiral Cu^II^‐bisoxazoline complex. The newly generated N‐centered radical is reduced by the previously formed Cu^I^ species of the first cycle to release the alkylated imine with high enantioselectivity.

## 3 Nickel

The use of Ni^II^‐^[20]^ or Ni^0^‐based^[21]^ complexes as standalone photocatalysts has only been sporadically reported.^[22]^ However, in the realm of metallaphotoredox‐catalyzed C−C cross‐coupling reactions, nickel compounds have been exploited most widely because of the excellent radical‐capturing ability (aryl, alkyl, acyl, etc.) and ligand lability of Ni^II^ species (d^8^ system). In this case, the formation of the products takes place either by oxidation‐induced reductive elimination from the electronic ground state of the Ni^III^ species^[23]^ or from excitation‐induced reductive elimination from an electronically excited state of Ni^II^* species.^[24]^

**Scheme 2:**
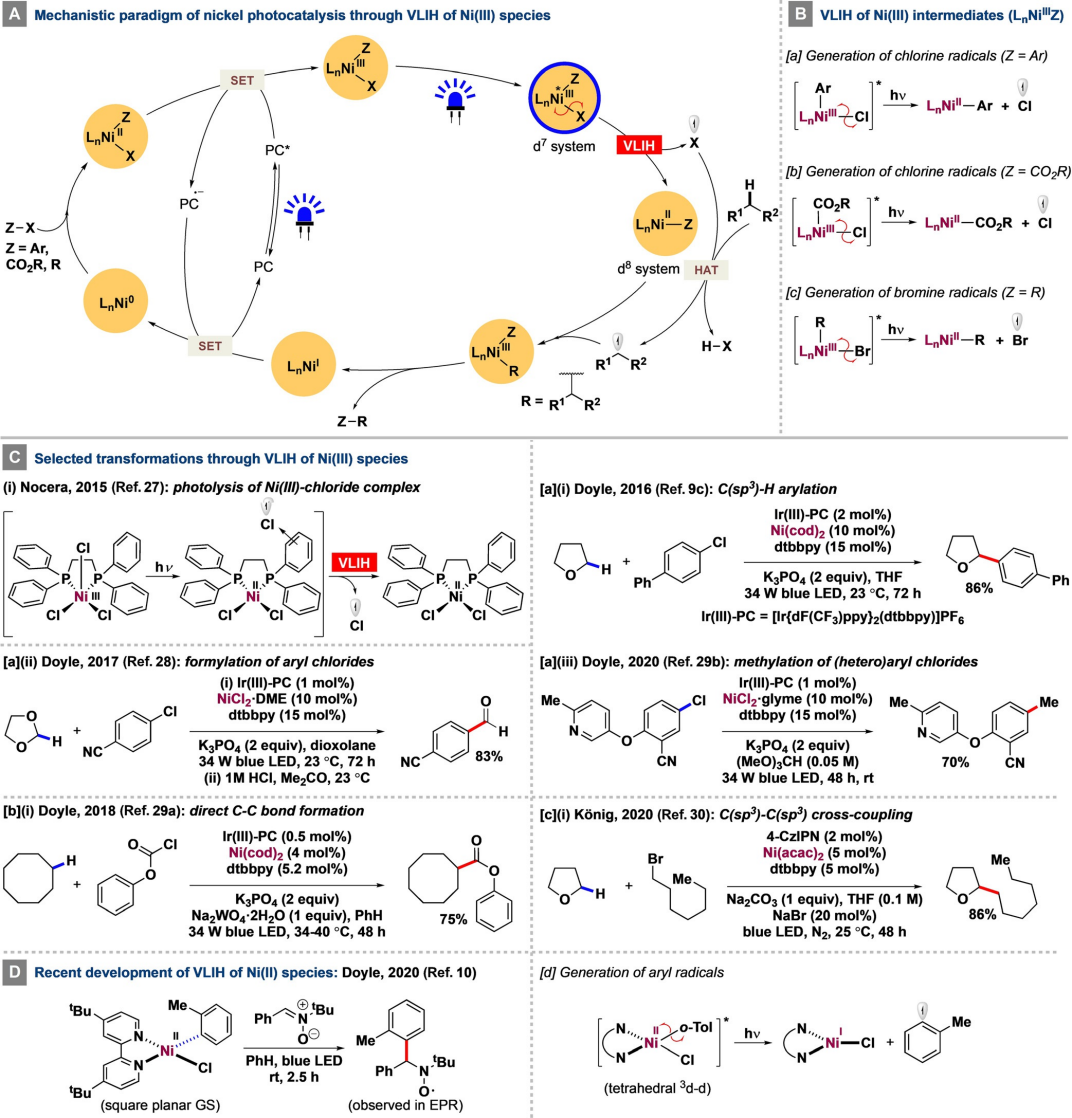
Mechanistic features of the VLIH of nickel species and selected transformations.

Halogen radicals can be generated from Ni^II^ complexes either by UV irradiation^[25]^ or through triplet‐triplet energy transfer from exogenous photocatalysts^[26]^ and used as HAT catalysts for C(sp^3^)−H cross‐coupling reactions. A counterintuitive mechanistic approach has emerged, wherein direct VLIH of high‐valent nickel(III) complexes is exploited to photogenerate halogen radicals (Scheme 2 B). In 2015, Nocera and co‐workers reported several Ni^III^ trihalide complexes from which homolytic photoextrusion of halogen radicals—intermediately stabilized by an arene‐to‐halogen‐atom charge‐transfer interaction in the secondary coordination sphere—could be possible from a dissociative LMCT excited state to induce a Ni−Cl σ→σ* transition [Scheme 2 C, Eq. i].^[27]^ The feature was subsequently exploited by the Doyle group in a series of cross‐coupling reactions involving the generation of alkyl radicals through C−H abstraction by the incipient photogenerated chlorine radical from light‐absorbing Ni^III^ species.[^9c^, ^28^, ^29^] The general mechanistic pathway initiates with the oxidative addition of L_*n*_Ni^0^ to the halide substrate to generate an intermediary ZL_*n*_Ni^II^X species, which is oxidized by the excited photocatalyst to give ZL_*n*_Ni^III^X species. Irradiation of this species with visible light results in the homolytic cleavage of the Ni^III^−X bond and generation of the corresponding halogen radical (X^.^) and Z‐L_*n*_Ni^II^ species. X^.^ can participate in a hydrogen atom transfer (HAT) process by interacting with the substrate (or a HAT mediator) to generate an incipient alkyl radical (R^.^), which gets trapped by the ZL_*n*_Ni^II^ species. The resulting ZL_*n*_Ni^III^R species can then undergo reductive elimination to furnish the cross‐coupled product Z‐R and a L_*n*_Ni^I^ species, which gets reduced by the reduced photocatalyst to L_*n*_Ni^0^ to complete both of the catalytic cycles (Scheme 2 A).

Doyle and co‐workers used this concept to develop a successful strategy for the (hetero)arylation of cyclic and acyclic ethers in the presence of [Ir(dF(CF_3_)ppy)_2_(dtbbpy)]PF_6_ as the exogenous photocatalyst and a Ni co‐catalyst, whereby (hetero)aryl chlorides were used as both the cross‐coupling partners and the chlorine radical source.^[9c]^ Although the strategy was effective for the abstraction of hydrogen from ethereal C(sp^3^)−H bonds [BDE(C‐H(THF))=92 kcal mol^−1^; Scheme 2 C, Eq. a‐i] by a chlorine radical generated through VLIH of [(dtbbpy)Ni^III^(aryl)(Cl)]‐type species and led to the formation of the corresponding benzylic ethers in up to 93 %, cyclohexane was only obtained in 41 % yield. The issue was addressed in a subsequent report by the same group, wherein chloroformates and acid chlorides were used as the cross‐coupling partner and the chlorine radical sources to functionalize C(sp^3^)−H bonds of unactivated alkanes (BDE=90–95 kcal mol^−1^) for the syntheses of various carbonyl derivatives [Scheme 2 C, Eq. b‐i].^[29a]^ A strategy for the alkylation of cyclic ethers has been developed by König and co‐workers, wherein VLIH of [L_*n*_Ni^III^(alkyl)(Br)] species (Scheme 2Bc) is involved in the generation of nascent bromine radicals that can act as HAT mediators and abstract hydrogen from ethereal C(sp^3^)−H bonds [Scheme 2 C, Eq. c‐i].^[30]^

In an expansionary study of the first report, Doyle and co‐workers also developed a selective formylation reaction of aryl chlorides by employing 1,3‐dioxolane as the solvent (instead of THF) and a post‐reaction mildly acidic workup of the reactions [Scheme 2 C, Eq. a‐ii].^[28]^ Here also, the key mechanistic step involves the generation of a chlorine radical by VLIH of [L_*n*_Ni^III^(aryl)(Cl)] species (Scheme 2Ba) to abstract hydrogen from the 2‐position of 1,3‐dioxolane (BDE(C2‐H)=86.8 kcal mol^−1^).

In the same line, the Doyle group reported a methylation strategy of (hetero)aryl chlorides using trimethyl orthoformate as the methyl radical source [Scheme 2 C, Eq. a‐iii].^[29b]^ The transformation proceeds via the formation and subsequent VLIH of [L_*n*_Ni^III^(aryl)(Cl)] species to generate an incipient chlorine radical that undergoes a HAT process with trimethyl orthoformate and a subsequent homolytic β‐scission to form the methyl radical for methylation.

A photophysical and photochemical study collaboratively conducted by the Castellano and Doyle groups on a series of (^R^bpy)Ni^II^(aryl)X‐type of complexes using ultrafast UV/Vis and mid‐IR transient absorption spectroscopy revealed that, upon irradiation with visible light, an initially formed square‐planar ^1^MLCT state of the complex gradually evolves over 5–10 ps into a long‐lived, tetrahedral ^3^d‐d (MC) state lying about 0.5 eV above the ground state with a lifetime of about 4 ns.^[10]^ This transition also results in a change in orbital symmetry to (e)^4^(t_2_*)^4^ and thus to a higher occupancy of antibonding orbitals (t_2_*) that weakens the Ni^II^−Ar bond and ultimately leads to its homolysis to generate the aryl radical (probed by spin‐trapping experiments with *N*‐*tert*‐butyl‐α‐phenylnitrone, Scheme 2 D) and a Ni^I^ species. The study refuted previously assigned long‐lived MLCT states^[31]^ and offered a new mechanistic pathway to initiate catalysis by Ni^I^. Indeed, an Ni‐catalyzed C‐O coupling strategy of (hetero)aryl electrophiles with 1° and 2° alcohols mediated by long‐wavelength UV light (*λ*=390–395 nm) was subsequently developed by Xue and co‐workers, wherein the photoexcited Ni^II^ intermediary complex undergoes Ni−C bond homolysis to generate aryl radicals and the Ni^I^ proceeds to take part in a Ni^I^‐Ni^III^ catalytic cycle to furnish the corresponding C−O cross‐coupled products.^[32]^

## 4 Iron

**Scheme 3:**
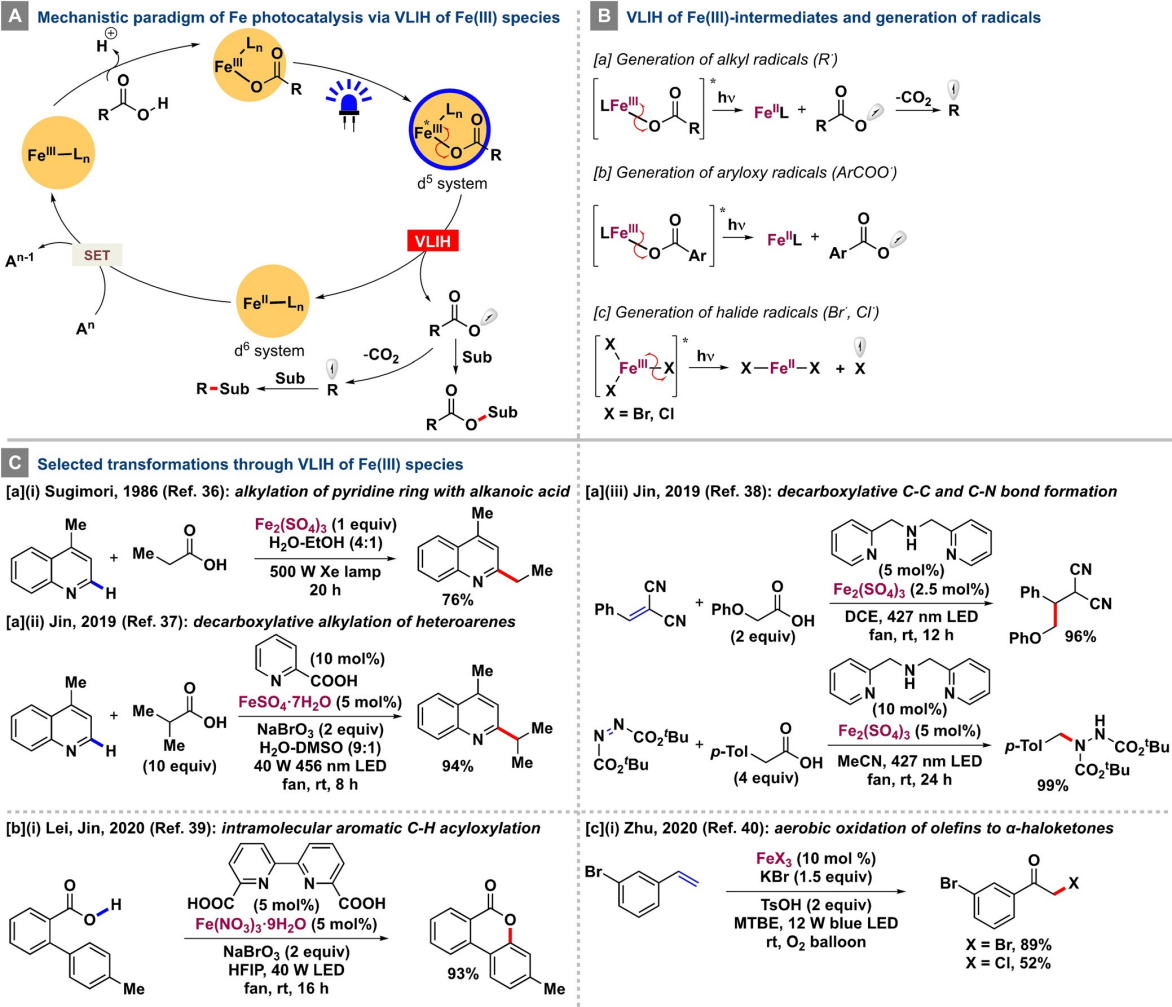
Mechanistic features of the VLIH of Fe^III^ species and selected transformations.

Unlike the precious‐element‐based (Ru^II^, Ir^III^, Os^II^, Re^I^, etc.) polypyridyl complexes, iron(II) complexes have found far fewer applications in organic photoredox catalysis because of their much shorter photoexcited‐state lifetimes. This arises because their MLCT excited states can be deactivated extremely rapidly (ca. 50 fs) by energetically lower‐lying metal‐centered (MC) excited states, which results in incompetent electron‐transfer reactivity and a lack of photoluminescence.^[33]^ Significant efforts have been expended to prolong the excited‐state lifetimes of iron complexes either by the use of chelating ligands that allow robust metal coordination to achieve high symmetry—ideally close to *O_h_* coordination—to maximize the overlap between the metal and ligand orbitals or by enhancing the ligand‐field strength, thereby raising the energy levels of the metal‐centered states by the use of ligands with strong σ‐donor and π‐acceptor properties such as N‐heterocyclic carbenes.^[34]^ However, another emerging complementary method entails in situ formation of photoactive iron–substrate complexes that can undergo VLIH to generate radicals that can initiate the desired reactions (Scheme 3 A).

Potassium ferrioxalate has been widely used as a sensitive chemical actinometer since the discovery of its photoinduced reduction to ferrous oxalate and carbon dioxide under irradiation at *λ*<490 nm, as first reported by Parker and Bowen in 1953.^[35]^ In 1986, Sugimori and Yamada reported that the alkylation of pyridine rings with alkyl radicals could be performed through the decarboxylation of alkanoic acids in the presence of visible light instead of γ‐rays by using ferric sulfate as a stoichiometric additive [Scheme 3 C, Eq. a‐i].^[36]^ The formation of a Fe^III^‐alkanecarboxylate complex that absorbed near‐ultraviolet visible light and could undergo VLIH followed by decarboxylation to generate the desired alkyl radical as well as the potency of Fe_2_(SO_4_)_3_ to act as an oxidant were postulated to facilitate the homolysis. Jin and co‐workers brought this transformation into the catalytic domain in 2019 with the successful photoinduced iron‐catalyzed decarboxylative alkylation of heteroarenes [Scheme 3 C, Eq. a‐ii].^[37]^ With the effective combination of 5 mol % FeSO_4_⋅7 H_2_O, 10 mol % 2‐picolinic acid as the ligand, and sodium bromate as the exogenous oxidant, a wide range of alkanoic acids and heteroarenes could be employed to furnish the corresponding products in up to 94 % yield. The key step involves the VLIH of a Fe^III^‐carboxylate complex to generate the Fe^II^ species and the carboxyl radical, which upon CO_2_ extrusion produces the nucleophilic alkyl radical (Scheme 3Ba). The exogenous oxidant oxidizes Fe^II^ to Fe^III^, which then reenters the catalytic cycle. In a subsequent report, the same group could extend the scope of the radical decarboxylative alkylation strategy by employing a range of Michael acceptors, such as alkylidenemalononitriles and azodicarboxylates, to furnish the corresponding products with C−C and C−N bonds, respectively.^[38]^ Of note, the electron‐deficient radical intermediate generated after the initial addition of the alkyl radical to the Michael acceptor could effectively oxidize Fe^II^ back to Fe^III^ to complete the catalytic cycle, thereby making the process redox‐neutral and obviating the use of any exogenous oxidant [Scheme 3 C, Eq. a‐iii].

After demonstrating the effectivity of iron(II) and iron(III)‐based photocatalysts in decarboxylative alkylation reactions, Lei, Jin, and co‐workers developed an intramolecular C−H oxygenation of 2‐biphenylcarboxylic acids in the presence of a catalytic amount of Fe(NO_3_)_3_⋅9 H_2_O/ 2,2′‐bipyridine‐6,6′‐dicarboxylic acid and two equivalents of sodium bromate as an exogenous oxidant under irradiation at *λ*=427 nm with an LED to synthesize several benzo‐3,4‐coumarins.^[39]^ The reaction proceeds through the intermediacy of an aryl carboxylate‐iron(III) complex which undergoes VLIH under the reaction conditions to furnish Fe^II^ and aroyloxy radicals that are almost impervious to decarboxylation at ambient temperature and could easily oxygenate aromatic C−H bonds. Subsequently, NaBrO_3_ can oxidize the Fe^II^ to Fe^III^ to complete the catalytic cycle [Scheme 3 C, Eq. b‐i].

Direct visible‐light‐induced homolysis of ferric halides has recently been exploited by Zhu and co‐workers when developing a straightforward and nonhazardous synthesis of α‐haloketones from activated olefins.^[40]^ The catalytic amount of FeX_3_ (X=Br, Cl) used in the reactions undergoes homolytic cleavage under irradiation with visible light to generate Fe^II^X_2_ and a halogen atom radical that gets trapped by the olefin. The resulting C‐centered radical reacts with oxygen and, upon dehydration, furnishes the desired product. An additional halogen source (KX) and TsOH assist in regenerating FeX_3_ to complete the catalytic cycle [Scheme 3 C, Eq. c‐i].

## 5 Cobalt

**Scheme 4:**
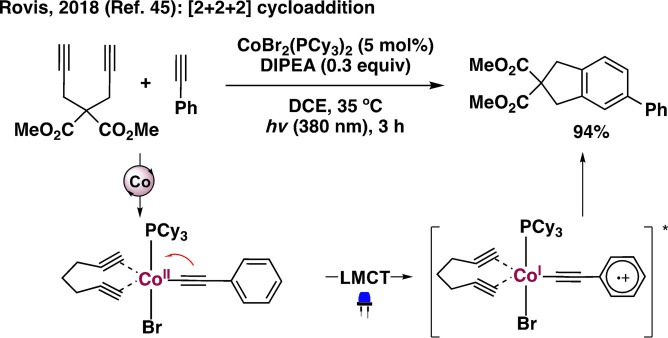
Photo‐ and Co‐acetylide‐catalyzed [2+2+2] cycloaddition reaction.

**Scheme 5:**
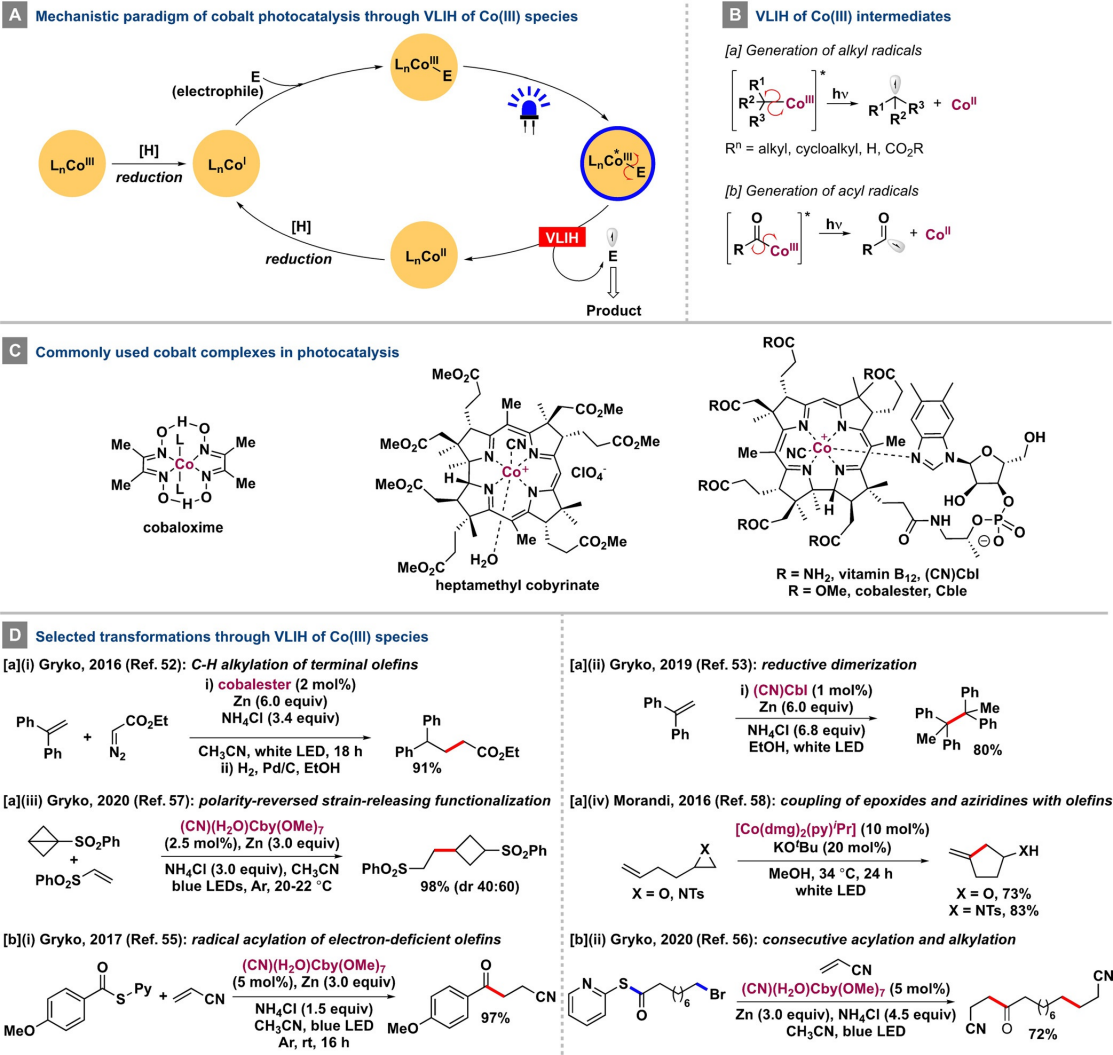
Mechanistic features of the VLIH of Co^III^ species and selected transformations.

Vitamin B_12_ (cobalamin), a naturally occurring organocobalt complex, has been utilized extensively in organic synthesis for its ability to undergo homolytic cleavage of the Co−C bond to generate C‐centered radical species.^[41]^ Different cobalt salts and complexes have been used in conjunction with exogenous photocatalysts to perform various dehydrogenative and C−C or C‐heteroatom bond‐forming transformations.^[42]^ Ligand photodissociation of CoH[PPh(OR)_2_]_4_‐type complexes is a well‐known feature^[43]^ that has been leveraged in various transformations.^[44]^ Nevertheless, VLIH of Co−R bonds is observed only when LMCT transitions are within the visible region and depends on the R moiety. The Rovis group has reported that in situ formed photoactive Co^II^‐acetylide species can undergo LMCT excitation upon irradiation (*λ*≈380 nm) to generate an aryl radical cation and a Co^I^ complex without any bond cleavage to assist in a subsequent oxidative cyclization process (Scheme 4).^[45]^ The distinctive catalytic activities of the three oxidation states of four‐coordinated cobalt complexes (“supernucleophilic” Co^I^, metalloradical Co^II^, organo‐, and hydro‐Co^III^ species) possessing substantial ligand field stabilization energy (LFSE) are characteristic features and are also involved in VLIH events (Scheme 5 A). Of note, several organo‐Co^III^ species are critical intermediates in cobalt‐catalyzed transformations involving photoinduced β‐hydride elimination^[46]^ as well as the VLIH process.^[47]^ In 2011, the Carreira group reported an intramolecular Heck‐type coupling of aryl iodides with olefins catalyzed by a cobaloxime complex, wherein the use of a mild base can deprotonate a hydridocobalt [Co^III^‐H] intermediate to regenerate the catalytically active Co^I^ species.^[48]^ Photoinduced homolysis of Co^III^−alkyl bonds was observed in alkylcobalamins and alkylcobaloximes under irradiation with a 100 W high‐pressure mercury lamp.^[49]^ In 2018, Soper and co‐workers carried out the trifluoromethylation of (hetero)arenes with [(^S^OCO)Co^III^(CF_3_)(MeCN)]‐type complexes supported by redox‐active [^S^OCO] pincer ligands.^[50]^ The trifluoromethylcobalt(III) complexes could undergo facile VLIH of the Co^III^−CF_3_ bond to release the corresponding Co^II^ species and a CF_3_ radical for further reactions. The resulting Co^II^ species can trap H^.^ to generate an unobserved [(^S^OCO)Co^III^(H)] intermediate that could produce H_2_ and regenerate the Co^I^ species to close the catalytic cycle. In 2019, Martin and co‐workers demonstrated an efficient approach for the activation of C−O bonds of alcohols by carbonylating them with Co^II^‐porphyrins to generate alkoxycarbonyl cobalt(III) complexes that could undergo VLIH of the Co−C bonds (BDE=39.8 kcal mol^−1^) and subsequent decarboxylation to furnish the corresponding alkyl radicals for trapping.^[51]^ A few common Co complexes are shown in Scheme 5 C.

In 2016, Gryko and co‐workers reported a cobalester‐catalyzed olefinic C(sp^2^)−H alkylation with diazo reagents as the carbene source.^[52]^ The key mechanistic step involves the VLIH of the alkylcobalester(III) species—formed by the reaction of Co^I^ with ethyl diazoacetate—to generate the Co^II^ and the corresponding α‐ester alkyl radical species for further transformation [Scheme 5 D, Eq. a‐i]. Co^I^ is regenerated by the reduction of the hydridocobalester (Co^III^‐H) intermediate. In 2019, the same group demonstrated the reductive dimerization of 1,1‐diphenylethylene in the presence of cobalamin‐like catalysts through VLIH of Co^III^−C bonds [Scheme 5 D, Eq. a‐ii] while studying the role of the nucleotide loop in general cobalamine‐catalyzed reactions.^[53]^

The generation of acyl radicals^[54]^ by the VLIH of Co^III^‐acyl complexes was also achieved by Gryko and co‐workers^[55]^ wherein the heptamethyl cobyrinate [(CN)(H_2_O)Cby(OMe)_7_], a vitamin B_12_ derivative, is initially reduced to the corresponding supernucleophilic Co^I^ complex that undergoes addition‐elimination with 2‐*S*‐pyridyl thioesters, the acyl radical precursors, to form the acyl‐vitamin B_12_ complex. Afterwards, VLIH of the Co−C bond furnishes the Co^II^ complex and the acyl radical that participates in the Giese‐type acylation of activated olefins [Scheme 5 D, Eq. b‐i]. In a subsequent study, the merger of the alkyl and acyl radical generation capabilities of the same Co^III^ catalyst through VLIH of Co−C bonds of in situ generated alkylcobalt(III) and acylcobalt(III) complexes was demonstrated, which allowed the consecutive Giese‐type alkylation and acylation of electron‐deficient olefins to synthesize highly functionalized molecules in a single step [Scheme 5 D, Eq. b‐ii].^[56]^ Of note, the in situ formation of the alkylcobalt(III) complex was faster than that of the acylcobalt(III) complex, which is reflected in the order of the two successive VLIH events in the reaction pathway.

A recent report from the Gryko group involves the visible‐light‐driven heptamethylcobyrinate‐catalyzed Giese‐type addition and Co/Ni‐catalyzed reductive cross‐coupling radical reactions of spring‐loaded cyclic reagents.^[57]^ The mechanistic pathway involves the initial formation of the Co^III^‐alkyl complex intermediate by the reaction of the “supernucleophilic” Co^I^ form of the catalyst and the electrophilic bicyclic reagents. The Co^III^‐alkyl complex subsequently undergoes visible‐light‐induced homolysis to generate the Co^II^ complex and the corresponding alkyl radicals that further engage in different radical transformations, such as the addition to SOMOphiles or transition‐metal‐catalyzed radical cross‐coupling reactions [Scheme 5 D, Eq. a‐iii].

A regioselective coupling reaction of epoxides and aziridines with alkenes in the presence of a simple cobalt dimethylglyoximate complex has been developed by Prina Cerai and Morandi to synthesize value‐added homoallylic alcohols and amines [Scheme 5 D, Eq. a‐iv].^[58]^ The key mechanistic steps of the transformation involve the nucleophilic opening of the epoxide/aziridine ring with Co^I^ and the VLIH of the Co^III^−C bond from the corresponding Co^III^ intermediate to generate Co^II^ and carbon‐centered radical species. The catalyst is regenerated with the help of the basic intermediate (e.g. alkoxide), which can deprotonate the Co^III^‐H species. The method successfully addressed the inefficiencies of the previous method reported by Harrowven and Pattenden by obviating the use of stoichiometric amounts of cobalt, base, and reductant.^[59]^

## 6 Cerium

Besides the ever‐increasing use of first‐row 3d transition‐metal complexes, interest has mounted substantially in recent times in the use of earth‐abundant lanthanide complexes. Being the 26th most abundant element, cerium has found extensive use in photocatalysis and warrants discussion in the context of VLIH. In a series of seminal studies conducted by Schelter and co‐workers, several luminescent cerium(III)‐based complexes have been used as both inner‐sphere^[60]^ and outer‐sphere potent single‐electron photoreductants.^[61]^ Their ability to absorb in the visible‐light region and undergo interconfigurational doublet‐to‐doublet, parity, and spin‐allowed 4f→5d metal‐centered electronic transitions, thereby minimizing the loss of energy from the long‐lived ^2^D excited states, provides a unique profile for application as photocatalysts.[^60^, ^61^, ^62^] Whereas the excited‐state Ce^III^ metalloradical (5d^1^) has been exploited for the abstraction of a chlorine atom from benzyl chlorides to generate benzyl radicals, the chloride‐Ce^IV^ LMCT excitation has also been leveraged in parallel photo‐oxidation processes involving C−C and C−heteroatom bond‐forming reactions.^[62d]^

**Scheme 6:**
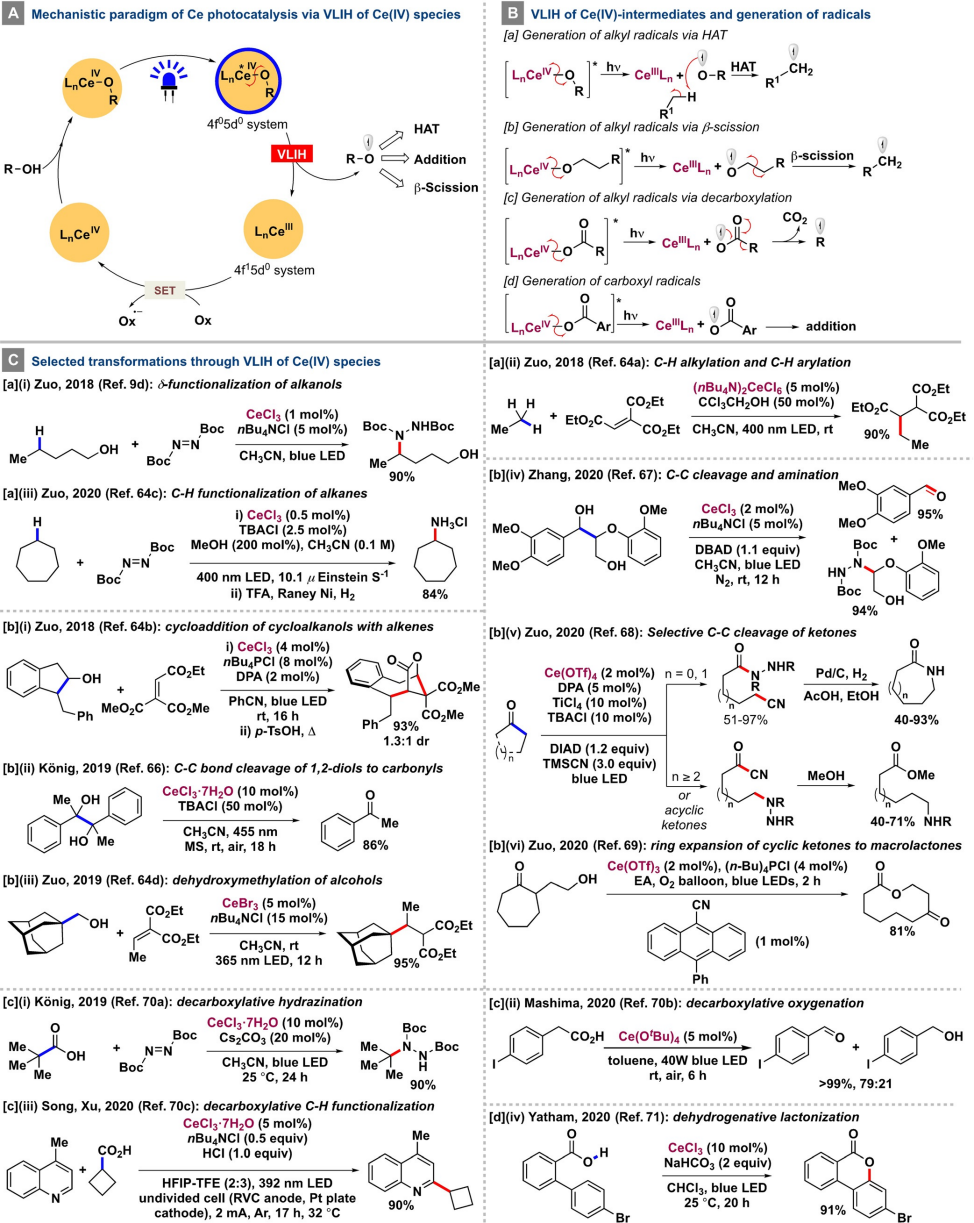
Mechanistic features of the VLIH of Ce^IV^ species and selected transformations.

The photocatalytic properties of Ce^III^ chloride complexes in a C−C bond‐cleavage and amination reaction of cycloalkanols was first reported by Zuo and co‐workers in 2016.^[63]^ However, a mechanistically different and complementary catalytic manifold of cerium photocatalysis was unveiled in a series of reports by the same group,[^9d^, ^64^] wherein the general mechanistic archetype involves: a) initial single‐electron oxidation of the Ce^III^L_*n*_ complex by an oxidant to generate an intermediary Ce^IV^L_*n*_ complex; b) coordination of an alcoholic ligand to form a L_*n*_Ce^IV^‐OR complex; and finally c) VLIH of the L_*n*_Ce^IV^‐OR species through photoinduced LMCT excitation and subsequent homolysis to generate oxygen‐centered radicals and Ce^III^L_*n*_ species to complete the catalytic cycle. The incipient, reactive alkoxide radical can then participate in a variety of transformations, such as intramolecular or intermolecular hydrogen atom abstraction (HAT), addition to another functional group, or homolytic β‐scission (Scheme 6 A).^[65]^

Based on these mechanistic features, Zuo and co‐workers developed a method for the efficient δ‐selective C−H bond functionalization of protecting‐group‐free primary alcohols in the presence of 1 mol % CeCl_3_ and 5 mol % *n*Bu_4_NCl.^[9d]^ In this process, the VLIH of the Ce^IV^‐OR complex under irradiation with visible light was carried out to generate a transient alkoxy radical that undergoes a thermodynamically favored intramolecular 1,5‐HAT to form a highly nucleophilic alkyl radical. Subsequent addition of this radical to DBAD and a SET reduction of the N‐centered radical furnishes the desired product and regenerates the Ce^IV^ catalyst [Scheme 6 C, Eq. a‐i]. The strategy of combining the VLIH of Ce^IV^‐OR complexes and intermolecular HAT was later expanded by the same group to valorize low molecular hydrocarbon feedstocks (C_*n*_H_2*n*+2_, *n*=1–4, Cy; BDE(C‐H)=105 kcal mol^−1^ for CH_4_) by successfully achieving C−H amination with DBAD, C−H alkylation with electron‐deficient alkenes, and Minisci‐type C−H heteroarylation [Scheme 6 C, Eq. a‐ii].^[64a]^ In 2020, Zuo and co‐workers extended this strategy to the C(sp^3^)−H functionalization of hydrocarbons [Scheme 6 C, Eq. a‐iii].^[64c]^ Of note, steady‐state homolysis experiments and transient absorption spectroscopic studies revealed that the VLIH event involving the Ce^IV^‐OMe complex was not the rate‐determining step in C−H amination and alkylation processes.

In a different mechanistic approach, an effective merger of the VLIH of Ce^IV^‐alkoxide complexes with a subsequent β‐C−C scission of the alkoxy radical species has been achieved to develop a range of useful transformations. In 2018, Zuo and co‐workers reported atom‐ and step‐economic formal cycloadditions of cycloalkanols with alkenes to afford bridged lactone scaffolds.^[64b]^ The key step of the developed transformation entails the VLIH of a Ce^IV^‐OR complex to generate a secondary alkoxy radical, which subsequently undergoes a rapid β‐scission process to form a nucleophilic alkyl radical that adds to an electron‐deficient alkene. Then, a SET process for the reduction of the generated α‐acyl radical by photoexcited 9,10‐diphenylanthracene (DPA; *E*_1/2_=−1.77 V versus SCE in CH_3_CN), an intramolecular aldol reaction of the enolate, and acidification furnishes the desired bridged lactone product [Scheme 6 C, Eq. b‐i]. When 1,2‐diols were employed as the substrate instead of alcohols, oxidative cleavage of the C−C bond was observed and the corresponding aldehydes were obtained in very high yields [Scheme 6 C, Eq. b‐ii].^[66]^ Following this approach, Zuo and co‐workers developed a dihydroxymethylation strategy wherein primary alcohols were converted into alkyl radicals with the loss of one molecule of formaldehyde, which underwent 1,4‐conjugate additions with Michael acceptors [Scheme 6 C, Eq. b‐iii].^[64d]^ Notably, a double‐excitation mechanism was proposed for the transformation, as it was observed that irradiation with LEDs at *λ*=365 nm could induce excitation of L_*n*_Ce^III^‐OR as well as ultraviolet‐induced homolysis (UVLIH) of L_*n*_Ce^IV^‐OR complexes, whereas a cerium/DPA dual photocatalytic system had to be employed under irradiation with LEDs at *λ*=400 nm as it could only effect the VLIH of the intermediary L_*n*_Ce^IV^‐OR complex.

Recently, Zhang and co‐workers successfully achieved the selective cleavage of C_α_−C_β_ bonds in various lignin model compounds in the presence of 2 mol % CeCl_3_ and 5 mol % *n*Bu_4_NCl under irradiation with visible light (*λ*=460 nm).^[67]^ The mechanistic pathway entails VLIH of the L_*n*_Ce^IV^‐lignin species coordinated through the benzylic α‐hydroxy group (α‐OH). The VLIH‐generated alkoxy radical intermediate enables cleavage of the C_α_−C_β_ bond to ultimately furnish the corresponding aldehydes (up to 97 %) and the hydrazinium derivatives (up to 95 %) by amination with DBAD [Scheme 6 C, Eq. b‐iv]. Zuo and co‐workers extended the activation strategy from alcohols to ketones through an effective merger of Ce^IV^‐VLIH and Lewis acid catalysis to selectively cleave C−C bonds of various acyclic and cyclic ketones and install different functional groups at the incipient acyl and alkyl radicals.^[68]^ The reaction proceeds through the activation of the carbonyl group by TiCl_4_ and nucleophilic addition of TMSCN to form the corresponding cyanohydrin derivative. Then, VLIH of the coordination complex formed between Ce^IV^ and cyanohydrin results in the formation of Ce^III^ species and an O‐centered radical species that undergoes β‐scission of the C−C bond to form a distal C‐centered radical—a process facilitated by the release of ring strain in the case of small cyclic ketones. Finally, orthogonal selective functionalization of both acyl and alkyl radicals with diisopropyl azodicarboxylate (DIAD) in the case of small and medium‐sized cyclic as well as acyclic ketones followed by a PET process with DPA furnish the desired products in high yields [Scheme 6 C, Eq. b‐v]. The same group has developed a straightforward ring‐expansion strategy of cyclic alkoxyketones to synthesize 9‐ to 19‐membered macrolactones in the presence of a cerium salt and cyanoanthracene under aerobic conditions through irradiation with visible light [Scheme 6 C, Eq. b‐vi].^[69]^ The successful development of the strategy relied upon the capability of the [Cl_*n*_Ce^IV^‐OR] intermediate to undergo VLIH to form [Cl_*n*_Ce^III^] and an alkoxy radical species for the subsequent β‐scission step. Of note, the mild Lewis acidic nature of the cerium salt was also conducive to enhance the formation of the lactol intermediate from the ketone/lactol tautomeric equilibrium. In a different set of transformations, effective decarboxylation of alkyl carboxylic acids has been achieved with Ce photocatalysis, wherein the key mechanistic step involves the VLIH of cerium‐carboxylate complexes of the Ce^IV^‐O(CO)R type to form Ce^III^ and highly reactive alkylcarboxyl radical species that undergo facile decarboxylation to generate the corresponding alkyl radicals for further transformations.^[70]^ In 2019, König and co‐workers utilized this property for the decarboxylative hydrazination of 1°, 2°, and 3° carboxylic acids in the presence of 10 mol % CeCl_3_⋅7 H_2_O and 20 mol % Cs_2_CO_3_ under irradiation with blue LEDs, wherein the corresponding VLIH‐generated alkyl radicals were trapped with DBAD to furnish the hydrazine derivatives in 28–90 % yield [Scheme 6 C, Eq. c‐i].^[70a]^ In 2020, Tsurugi, Satoh, Mashima, and co‐workers performed the decarboxylative oxygenation of aliphatic carboxylic acids in the presence of 5 mol % Ce(O^*t*^Bu)_4_ under air at atmospheric pressure to obtain products containing C−O bonds such as aldehydes and ketones in up to quantitative yields.^[70b]^ The transformation proceeds via the formation of a hexanuclear oxocerium(IV) carboxylate complex cluster, Ce_6_O_4_(OH)_4_(OCOR)_12_, from the reaction between Ce(O^*t*^Bu)_4_ and carboxylic acids. This hexanuclear Ce^IV^ species undergoes VLIH under irradiation with blue light and forms Ce^III^ species and a carboxyl radical that further engages in decarboxylation and oxygenation to form the corresponding alkyl peroxyl radical (RCH_2_OO^.^). This radical forms alkylperoxo‐Ce^IV^ species, from which alkyl hydroperoxides are formed that undergo dehydration to finally afford the corresponding aldehydes as the terminally oxidized major products along with minor amounts of the corresponding alcohols [Scheme 6 C, Eq. c‐ii].

Recently, Song, Xu, and co‐workers have brought the decarboxylative alkylation of heteroarenes with aliphatic carboxylic acids and cerium into the electrophotocatalytic domain,^[70c]^ wherein the reaction is initiated by the anodic oxidation of Ce^III^ to Ce^IV^, which coordinates with the carboxylic acid. A subsequent VLIH of the Ce^IV^‐carboxylate complex and decarboxylation forms the corresponding alkyl radical that adds to the heteroarene in a Minisci‐type reaction to afford the alkylated product in good to high yields [Scheme 6 C, Eq. c‐iii]. A photocatalytic method for the dehydrogenative lactonization of 2‐arylbenzoic acids in the presence of CeCl_3_ as the photocatalyst and O_2_ as the terminal oxidant has been developed by Yatham and co‐workers.^[71]^ In this process, a Ce^IV^‐aryl carboxylate complex is formed by the coordination of the aryl carboxylic acid with Ce^IV^, and subsequent VLIH generates a Ce^III^ species and the corresponding aryl carboxyl radical, which gets trapped by the aryl substituent without undergoing decarboxylation and eventually furnishes the lactonized product in very high yields [Scheme 6 C, Eq. c‐iv].

## 7 Miscellaneous Examples

Although not explored for synthesis in as much detail as the specific cases discussed previously, there is increasing recognition of the potential to develop photocatalytic transformations with other transition‐metal‐based photocatalysts such as vanadium, chromium, manganese, or palladium by capitalizing on their ability to undergo VLIH. VLIH of Mn‐alkyl bonds has been studied with various Mn(CO)_5_R‐type complexes.^[72]^ Photoinduced homolytic cleavage of a range of paramagnetic Cr^III^ monohydrocarbyl complexes with the general formula CpCr[(ArNCMe)_2_CH](R) has also been studied in detail.^[73]^

**Scheme 7:**
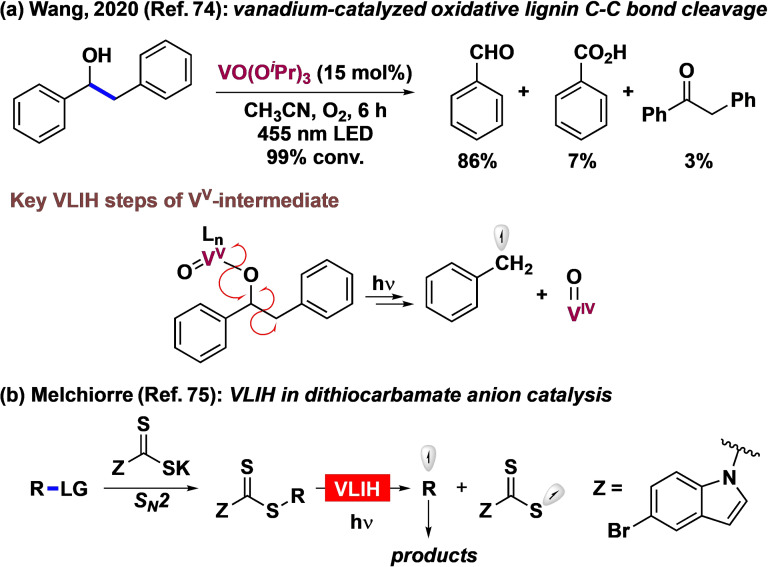
Recent developments in VLIH: a) Vanadium‐catalyzed oxidative C−C cleavage of lignin and the mechanistic features; b) VLIH in catalysis with a dithiocarbamate anion.

Wang and co‐workers have recently reported vanadium(V)‐catalyzed visible‐light‐driven selective C_α_−C_β_ bond cleavage of β‐1 interlinkages of lignin models to afford valuable aromatic products (Scheme 7 a).^[74]^ The proposed mechanistic pathway for this transformation entails the initial coordination of the benzylic hydroxy group to the vanadium center. Then, excitation of the resulting complex with visible light induces an LMCT process and reduction of the vanadium center, which in turn causes the homolytic cleavage of the C_α_−C_β_ bond to produce benzaldehyde and a benzyl radical for further reaction. Torres et al. have reported a visible‐light‐driven palladium‐catalyzed carbonylation reaction to synthesize acid chlorides from aryl halides, wherein the irradiation with light assists in the initial radical‐induced oxidative addition of Pd^0^ as well as excitation of the Pd^II^ intermediate that subsequently undergoes photoinduced reductive elimination.^[75]^ Interestingly, one of the possible pathways for the last mechanistic step involves VLIH of a Pd−acyl bond to generate the incipient acyl radical in the reaction medium, which has been probed by trapping experiments.

The VLIH concept has also been successfully applied to organic catalyst–substrate complexes. Melchiorre and co‐workers recently used a nucleophilic dithiocarbamate anion catalyst with an attached chromophoric unit to activate various alkyl electrophiles bearing different leaving groups through an S_N_2 pathway. The resulting photon‐absorbing intermediate undergoes VLIH to generate C‐centered radicals which can, thereafter, participate in various C−C bond‐forming reactions (Scheme 7 b).^[76]^

## 8 Summary and Outlook

As can be gleaned from the examples discussed in this Minireview, by utilizing the VLIH concept with earth‐abundant transition‐metal‐based photocatalysts it is possible to transcend the limits of traditional photocatalysts that demonstrate only outer‐sphere electron‐transfer and energy‐transfer processes from their excited, populated, and emissive MLCT states. The concept has also been successfully applied within the wavelength range of 350–400 nm, where the event could be termed as UVLIH. Although the majority of the VLIH processes that have found successful applications in organic synthesis involve dissociative LMCT of different metal–substrate complexes to generate targeted radicals, evermore variants of electronic transitions such as dissociative ^1^MLCT/^3^d‐d or dissociative SBLCT transitions are also being recognized, together with the continuous advancement in the field of sophisticated spectroscopic methods and computational studies to determine the intermediate radicals species and complexes. With the creative exploitation of the VLIH activation mode, new synthetic processes are possible, wherein the reaction pathways will be directed by the inner‐sphere mechanism of the sustainable photocatalysts and should, in turn, allow the development of enantioselective approaches and the generation of new radical species by the selective homolysis of metal–substrate bonds. We are confident that alternative modes and applications will be discovered through the effective collaboration of synthetic organic chemists and spectroscopists, and that the so far discovered methods will find wide applications in both academic and industrial set‐ups.

## Conflict of interest

The authors declare no conflict of interest.

## Biographical Information

*Youssef Abderrazak was born in Casablanca, Morocco. He obtained his M.Sc. in organic chemistry from the Hassan II University of Casablanca. His M.Sc. thesis was conducted under the supervision of Prof. J. Jamaleddine on the synthesis of pyrans by organocatalysis. He then joined the group of Prof. O. Reiser at the University of Regensburg, where he is doing his Ph.D. His research interests are photocatalysis and synergistic catalysis*.

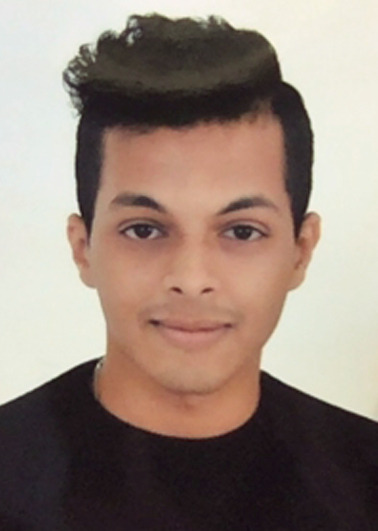

## Biographical Information

*Aditya Bhattacharyya was born in West Bengal, India. He graduated in Chemistry from the University of Calcutta and received his M.Sc. from Bengal Engineering and Science University, Shibpur. Subsequently, he obtained his Ph.D. in organic chemistry under the supervision of Prof. M. K. Ghorai at the Indian Institute of Technology, Kanpur. He then moved to the group of Prof. O. Reiser at the University of Regensburg with a Marie Skłodowska‐Curie Individual Fellowship to pursue postdoctoral studies in the field of photoredox catalysis*.

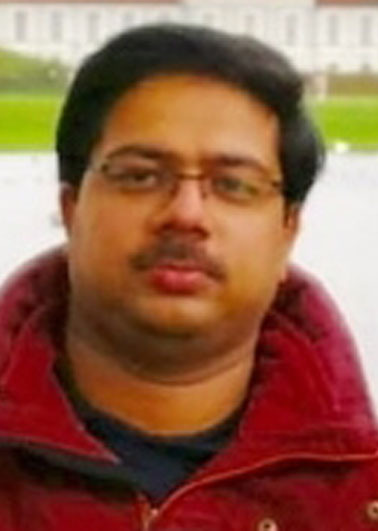

## Biographical Information

*Oliver Reiser studied in Hamburg, Jerusalem, and at UCLA, obtaining his Ph.D. under the supervision of Prof. A. de Meijere. After postdoctoral research at IBM with Dr. R. D. Miller and at Harvard University with Prof. D. A. Evans, he began his independent career at the University of Göttingen. He moved to Stuttgart as an associate professor and became a full professor of organic chemistry in 1997 at the University of Regensburg. His research interests center around synthesis and catalysis, including the development of new photo‐ and magnetic nanoparticle catalysts*.